# Polystyrene-Based Nanocomposites with Different Fillers: Fabrication and Mechanical Properties

**DOI:** 10.3390/polym12112457

**Published:** 2020-10-23

**Authors:** Olga A. Moskalyuk, Andrey V. Belashov, Yaroslav M. Beltukov, Elena M. Ivan’kova, Elena N. Popova, Irina V. Semenova, Vladimir Y. Yelokhovsky, Vladimir E. Yudin

**Affiliations:** 1 Ioffe Institute, 26, Polytekhnicheskaya, 194021 St.Petersburg, Russia; olga-moskalyuk@mail.ru (O.A.M.); belashov.andrey.93@gmail.com (A.V.B.); ybeltukov@gmail.com (Y.M.B.); 2 Petersburg State University of Industrial Technologies and Design, 18, Bolshaya Morskaya str., 191186 St. Petersburg, Russia; 3 Institute of Macromolecular Compounds, 31, Bolshoy pr. V.O., 199004St. Petersburg, Russia; ivelen@mail.ru (E.M.I.); men682003@mail.ru (E.N.P.); vlad_elokhovskiy@bk.ru (V.Y.Y.); yudin@hq.macro.ru (V.E.Y.)

**Keywords:** polymer nanocomposites, polystyrene, elastic moduli, strain solitons, digital holography

## Abstract

The paper presents a comprehensive analysis of the elastic properties of polystyrene-based nanocomposites filled with different types of inclusions: small spherical particles (SiO_2_ and Al_2_O_3_), alumosilicates (montmorillonite, halloysite natural tubules and mica), and carbon nanofillers (carbon black and multi-walled carbon nanotubes). Block samples of composites with different filler concentrations were fabricated by melt technology, and their linear and non-linear elastic properties were studied. The introduction of more rigid particles led to a more profound increase in the elastic modulus of a composite, with the highest rise of about 80% obtained with carbon fillers. Non-linear elastic moduli of composites were shown to be more sensitive to addition of filler particles to the polymer matrix than linear ones. A non-linearity modulus *β_s_* comprising the combination of linear and non-linear elastic moduli of a material demonstrated considerable changes correlating with those of the Young’s modulus. The changes in non-linear elasticity of fabricated composites were compared with parameters of bulk non-linear strain waves propagating in them. Variations of wave velocity and decay decrement correlated with the observed enhancement of materials’ non-linearity.

## 1. Introduction

Micro- and nano-structured composites have become very popular nowadays in various engineering applications, see e.g., [1]. Most of them contain ordered inclusions, i.e., they are manufactured in the form of a matrix filled with oriented filaments made of another material. Numerous examples can be mentioned in this context, from rebar-reinforced concrete to carbon- and boron-reinforced composites used in solar batteries. Disordered composites are of common interest as well, with the most intriguing ones being filled with nanoparticles, which can drastically improve the resulting material strength, stiffness and other physical parameters, providing at the same time a uniform material at the macroscale. Numerous examples have been published recently demonstrating significant advantages of nanocomposites over matrix materials, see e.g., [2,3,4,5,6,7,8,9,10,11], in particular in view of their enhanced mechanical properties and potential multifunctionality. Composites on the base of polymer matrices with various nano-sized fillers are one of the most widely used classes of nanocomposites.

However, in view of mechanical properties a major disadvantage, common for all polymer nanocomposites, is low predictability of resultant parameters. The uncertainty is mainly due to non-uniform distribution and agglomeration of filler particles in a matrix causing areas with reduced interfacial interaction between the matrix and the filler. The existing experimental data demonstrate considerable variations of measured parameters for the same compositions of materials [8].

In the first approximation, elastic properties of a material are governed by linear elastic moduli, which characterize elastic stresses of a solid at small strains. Since linear moduli give a quadratic contribution to the elastic energy, they are also called the second-order moduli. Linear elastic properties of composite materials have been extensively studied both in theory and experiments and expressions for second-order moduli of elasticity have been derived in various models, see e.g., [12,13]. It is worth noting, however, that at comparatively low volume concentrations (up to 10–15%) of a filler the effective moduli of a composite calculated in terms of the aforementioned models give close values. However, theoretical estimations of elastic moduli values are, as a rule, much higher than the results of measurements. It may be due to an applied assumption of regular (uniform) distribution of nanoinclusions in a polymer, that often is not found in real materials. Inhomogeneous distribution of nanoinclusions in a matrix and their micron-sized agglomerates lead to inhomogeneous strength in the composite volume and appearance of local finite-size areas with lower stiffness. For this reason, the micromechanical models were refined to take account for intrinsic small-scale inhomogeneities and their impact on the structural strength, see, e.g., [14,15,16].

At higher strains non-linear elasticity makes an increasingly notable contribution to material behavior. The deviation of stress-strain relation in elastic regime from linear dependence is described by third-order (non-linear) elastic moduli. In Murnaghan’s theory [17], the non-linear elastic behavior of an isotropic solid material is described by three non-linear, third-order moduli (*l*, *m*, *n*) and two linear, second-order, Lamé constants (*λ*, *μ*). The third-order elastic moduli and their linear combinations are informative for the prediction of fatigue damage, for description of thermoelastic properties of crystalline solids, acoustic radiation stress, radiation-induced static strains, creep, thermal aging, wave processes, etc. In general, non-linear parameters were shown to be more sensitive to structural changes in a material than linear ones.

In nanocomposites, the non-linear elastic effects are enhanced due to localization of deformation near nanoparticles. Theoretical models of the non-linear elastic properties of composite materials are still in active development [18,19,20,21,22,23]. A theory accounting for non-linear elastic properties of both the matrix and the filler was developed recently for the case of spherical inclusions [24]. The experimental validation of theoretical predictions is highly desirable, however now measurements of non-linear elastic properties of composites are very rare. To the best of our knowledge no data on that for polymeric composites has been published as yet.

A wide variety of existing nanofillers can be classified based on their dimensionality: 2D (nanosheets), 1D (nanotubes), and 0D (spherical nanoparticles) [6]. As is known, carbon-based nanofillers exhibit most promising properties as potential nanofillers due to their high mechanical strength and high aspect ratio. For instance, carbon nanotubes were shown to have tensile modulus up to 1 TPa, tensile strength in the range of 50–150 GPa [25,26] and high aspect ratio (>1000). Among 2D nanofillers, montmorillonite sheets provide relatively high Young’s modulus reported to be within the range of 178–265 GPa [27] and relatively high aspect ratio (>50). The excellent properties of these nanofillers make them good candidates for reinforcement of polymer matrices. However, the major drawback of high-aspect-ratio particles is an elevated agglomeration due to an increased surface-to-bulk ratio causing forces that attract particles to each other. Zero-dimensional particles demonstrate much lower agglomeration but provide moderate enhancement of mechanical properties.

In this paper we report a complex multi-method experimental analysis of mechanical properties of composites based on a polystyrene matrix with addition of nanofillers of different nature, dimensionality and size. Composite samples with different filler concentrations were fabricated by melt technology, and properties of both composite melts and resulting composite samples were examined and analyzed.

The paper is organized as follows. Section 2 is devoted to the description of the applied materials, composite fabrication technology and methodologies used for testing of composite samples. The results obtained and the discussion are presented in Section 3. First we describe properties of composite melts obtained from thermal analysis by differential scanning calorimetry and control of filler distribution in the polymer matrix. Then we present data on linear elastic properties (elastic modulus, strength and strain at break) of composites obtained from tensile tests. We focus on those composites which demonstrated most profound changes of linear parameters as compared to pure polymer and present data on their non-linear elastic parameters obtained from ultrasonic measurements at static stress. Finally, we analyze changes in composites elasticity on the specific example of non-linear elastic process: evolution of bulk strain solitary waves in fabricated materials. The conclusions made are summarized in Section 4.

## 2. Materials and Methods

### 2.1. Materials

Grained 585 polystyrene (PS) (Nizhnekamskneftekhim, Nizhnekamsk, Tatarstan, Russia) was used as a polymer matrix for all the composite samples. The main parameters of PS as specified by the manufacturer are shown in Table 1. The following materials were applied as (nano)fillers: Silicon dioxide (SiO_2_) particles Aerosil R812 modified by silazane (Evonic Industries AG, Essen, Germany); Alumina nanoparticles (Al_2_O_3_) Aeroxide Alu C805 modified by octylsilane (Evonic Industries AG, Essen, Germany); carbon black (CB) P-805E (Ivanovskiy tekuglerod and rubber, Ivanovo, Russia), multi-walled carbon nanotubes (CNT) CTube-100 (N Co. Ltd., Songdo-dong Yeonsu-gu Incheon, Republic of Korea); halloysite natural tubules (HNT) (NaturalNano Inc., Rochester, NY, USA), Sheet silicate (phyllosilicate) minerals Mica ME-100 (Mica) (CBC Co Ltd., Tokyo, Japan), Montmorillonite 15A (MMT) (Southern Clay Products Inc., Houston, TX, USA). The data on filler particle sizes are summarized in Table 2.

Mechanical properties of polymer composites were shown to be noticeably improved with an increase in the axial ratio of nanoparticles [28,29], but this holds true at low filler concentrations. For spherical dispersed particles an increase in strength or elastic modulus of polymer composites was observed at filler concentrations even over 20% [30]. An introduction of carbon nanotubes provided the increase of strength and stiffness of composites at a few percent or even tenths of a percent [31,32]. Layered silicates with individual nanolayers of about 1 nm thick provided significant improvement of material properties at low concentrations [33,34,35]. Therefore, in this work, spherical particles were introduced into the PS matrix at up to 20% with regard to polymer weight, layered silicates at up to 5% wt., and anisodiametric filler concentrations did not exceed 10–15% wt.

Nanocomposites consisting of the PS matrix with specified nanofillers were manufactured by melt technology. Its affordability and efficient performance frequently make this technology a method of choice for polymer processing in various industries. PS-based compositions were prepared using a twin-screw micro compounder DSM Xplore 5 mL (Xplore Instruments BV, Sittard, Netherlands). Compounding was carried out at 220 °C for 10 min at 50 rpm/min. Block samples were fabricated by injecting the solution into a die heated to 80 °C. When removed from the microinjector the die self-cooled to room temperature in air. Block samples of composites of two types have been manufactured: plates 50 mm × 10 mm × 1.5 mm and blades with the work area of 20 mm × 4 mm × 1.5 mm. Reference samples of the same shapes but made of pure PS have been manufactured as well for reference.

### 2.2. Testing of Composite Samples

As known, several key factors are critical for achieving an improvement in mechanical properties of a polymer through addition of a nanofiller. The following requirements should be addressed: (1) filler particles should have mechanical properties notably different from those of the matrix; (2) it is preferable that they would have high aspect ratio and high surface area to enable better interaction with the polymer; and (3) they should be well dispersed and agglomeration should be avoided [6].

Comprehensive analysis was undertaken on the mechanical properties of both PS-filler melts and composite samples. Rheological characteristics of melts of PS with different kinds of particles were determined using the rheometer Physica MCR 301 (Anton Paar GmbH, Graz, Austria) in a CP25-2 cone-plane measurement unit at 200 °C and 220 °C in shear and dynamic (oscillatory) modes with a decrease (Down) and increase (Top) of the strain rate (circular frequency) in air. The influence of particle concentration on the mechanical properties of nanocomposites in tension was studied using an Instron 5940 (Instron, Norwood, MA, USA) universal testing system at a stretching rate of 5 mm/min and base length of 20 mm. Based on the tensile test data, the strength at break (*σ*_b_, MPa), strain at break (*ε*_b_, %) and elastic modulus (*E*_0_, GPa) have been determined. The dispersion of nanoparticles in the polymer matrix was estimated from the micrographs of cryo-cleaved surfaces of the composite samples. The micrographs were taken using a Carl Zeiss Supra-55 scanning electron microscope (Carl Zeiss AG, Oberkochen, Germany).

### 2.3. Evaluation of Non-Linear Elastic Properties

Non-linear elastic properties of composite samples were examined through relative variations of the third-order elastic moduli in nanocomposite samples as compared to those of pure polymer samples. The experimental methodology is based on the approach suggested by Hughes and Kelly [36] and utilizes the analysis of the dependence of the velocity of longitudinal and shear ultrasonic waves in the sample upon the applied transverse static stress. See [37] for details of the applied methodology.

Specimens were composed of 3 plates of a composite adhesively bonded with the Superglue ethylcyanoacrylate adhesive. Uniaxial static stress within the range of 0–20 MPa was applied to a bigger side of the rectangular specimen. Testing was performed in perpendicular direction by longitudinal and shear sine ultrasonic waves at 2.25 MHz. Three sets of experiments were performed for each sample and velocities of three types of ultrasonic waves were measured as a function of applied static stress: (1) longitudinal waves, (2) shear waves parallel to the direction of applied stress, and (3) shear waves perpendicular to that. A convolution of the sinusoidal signals obtained with and without stress was calculated for each stress value. The delay time difference, introduced by variation of the ultrasonic wave velocity under applied stress, was estimated by calculation of the time point, corresponding to the convolution maximum. Wave shifts were recorded at a stepwise increase of the applied static stress that gave data on changes of wave velocity as a function of stress. Then the set of introduced effective longitudinal and shear moduli: *M_x_ = V_x_^2^ρ*_0_, *G_y_ = V_y_^2^ρ*_0_, *G_z_ = V_z_^2^ρ*_0_, plotted as function of stress provided data on the set of second- and third-order elastic moduli of the specimen material. The second-order Lamé moduli *λ*, *μ* were calculated from wave velocities at zero stress and third-order Murnaghan moduli *l*, *m*, *n* from the slope coefficients, as shown in [37].

The resulting equations for calculation of the set of Murnaghan moduli can be written as:

(1) $$l=-\frac{3\lambda +2\mu }{2}\alpha_{x}-\frac{\lambda (\lambda +\mu )}{\mu }\left(1+2\alpha_{y}\right)+\frac{\lambda^{2}}{2\mu }\left(1-2\alpha_{z}\right)$$

(2) $$m=-2\left(\lambda +\mu \right)\left(1+\alpha_{y}\right)+\lambda \left(1-\alpha_{z}\right)$$

(3) $$n=-4\mu \left(1+\alpha_{y}-\alpha_{z}\right)$$

where *α_x_,* *α_y_* and *α_z_* are dimensionless slope coefficients of the dependencies of corresponding effective moduli as function of applied uniaxial stress.

As known, the non-linear elastic moduli are more sensitive to structural changes in a material than the linear ones. Obviously, the third-order moduli of composites depend drastically not only on the type and concentration of inclusions but also on their distribution in the matrix. Inhomogeneous distribution and formation of agglomerates can cause considerable changes in these parameters. That means that, ideally, measurements of these parameters should be taken for each particular composite sample.

### 2.4. Generation and Monitoring of Non-Linear Strain Waves

The non-linear elastic properties of materials can provide conditions favorable for formation of bulk strain solitary waves in waveguides made of them. As known ([38]), the non-linear elasticity of a material can cause formation of a longitudinal strain wave *u* with the amplitude *A*, velocity *v* and width *L*, that is described by the equation:

(4) $$u\left(x-vt\right)=Acosh^{-2}(\frac{x-vt}{L})$$

It can be shown that the relationship of the soliton amplitude with its velocity depends only on integral parameters of the material and takes the form:

(5) $$A=3\left(v^{2}-c^{2}\right)\frac{\int \rho dydz}{\int \beta_{s}dydz}$$

where *c* is sound velocity and *β*_s_ < 0 is the nonlinearity modulus comprising a combination of the second- (*E*, *ν*) and third-order Murnaghan (*l*, *m*, *n*) elastic moduli of the material [38]:

(6) $$\beta_{s}=3E+2l{\left(1-2\nu \right)}^{3}+4m{\left(1+\nu \right)}^{2}\left(1-2\nu \right)+6n\nu^{2}$$

In Equation (5), a possible dependence of *ρ* and *β*_s_ on the transverse coordinates y and z is taken into account. In our experiments, solitons were formed in bar-shaped waveguides from initial laser-induced shock waves generated in water nearby the waveguide input. Soliton evolution in a waveguide was monitored using a digital holographic set-up as described in detail in our earlier papers, e.g., [39,40]. Recording of off-axis digital holograms by a high-speed global-shutter camera Nanogate 24 (Nanoscan, Moscow, Russia), providing exposure time of 100 ns, allowed us to detect long strain solitary waves, propagating at the velocity of about 2000 m/s. Large field of view (5 cm in diam.) of the implemented holographic setup enabled registration of entire smooth trough-shape waves with full width at half maximum (FWHM) of about 3 cm in a single frame. The holographic approach provides information on spatial distributions of refractive index gradient inside a transparent sample and was already demonstrated to be an efficient tool for detection of local density/thickness variations formed by a strain wave (see [39,41] and references therein). Since propagation of longitudinal strain waves in a transparent waveguide results in its local deformation, wave front shape of the recording laser is affected by local soliton-induced increase of waveguide thickness, which is monitored by a holographic technique. Soliton amplitude and width were determined from the obtained phase shift distributions and its velocity was calculated from precise measurements of its position in the course of propagation in the waveguide. Note that the reported holographic arrangement operates with transparent specimens only. To perform wave detection in opaque materials, as all composites are, we recently suggested an approach allowing for indirect recording of strain waves in opaque materials by monitoring phase shift gradients in a layer of transparent material adhesively bonded to the layer made of the material of interest [42,43]. As shown in [42], in a layered bar made of two different materials a single soliton is formed, the amplitude and width of which depend upon elastic properties of the corresponding materials. As shown in [43] the soliton velocity measured in such a sandwich waveguide in one of the layers equals the arithmetic mean of soliton velocities in waveguides of the same geometry but made of each of the materials.

Due to the integral dependence of soliton amplitude and velocity on the waveguide parameters (Equation (5)) these characteristics do not depend on the number and order of longitudinal layers of different materials in a waveguide. That is why in experiments we used two- and three-layered bars 10 mm × 10 mm in cross-section, made of a transparent layer of commercial PS and a layer(s) of a fabricated PS-based nanocomposite. Measurements made with these waveguides were compared with those made with a similar waveguide where the nanocomposite layer was substituted by a layer of pure PS but fabricated by the same technology as the composite. The nanocomposite layers and those of fabricated PS samples were made by bonding the corresponding plates with the ethylcyanoacrylate Superglue adhesive. As we have demonstrated previously [43,44], elastic features of this adhesive are close to those of the applied polymers and it does not affect noticeably the soliton behavior.

In these experiments we tested nanocomposite samples with the fillers of three types: spherical particles SiO_2_, alumosilicate particles with high aspect ratio HNT, and carbon particles CB. Evolution of solitons in the waveguides with these materials was monitored and their velocities and decay decrements were determined.

## 3. Results and Discussion

### 3.1. Properties of Composite Melts

**Figure 1:**
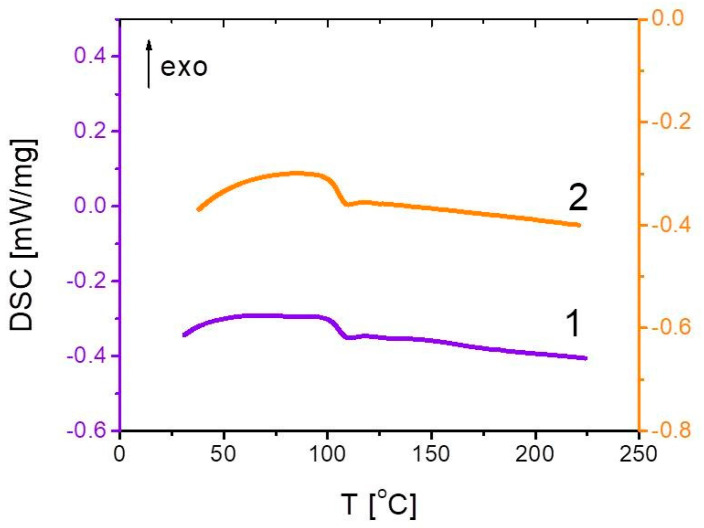
Differential scanning calorimetry (DSC) curve of pure polystyrene (PS) pellets (1st and 2nd scan).

Temperature ranges optimal for polystyrene processing by melt technology, i.e., temperature in the extruder chamber and mold temperature, were evaluated by thermal analysis by differential scanning calorimetry (DSC) using DSC 204 F1 (NETZSCH-Gerätebau GmbH, Selb, Germany). The glass transition temperature of pure PS pellets obtained from DSC thermograms (Figure 1) comprised *T*_g_ = 106 °C. Therefore the temperature range for polymer processing was chosen to be 200–220 °C, while the mold temperature had to be below 100 °C.

**Figure 2:**
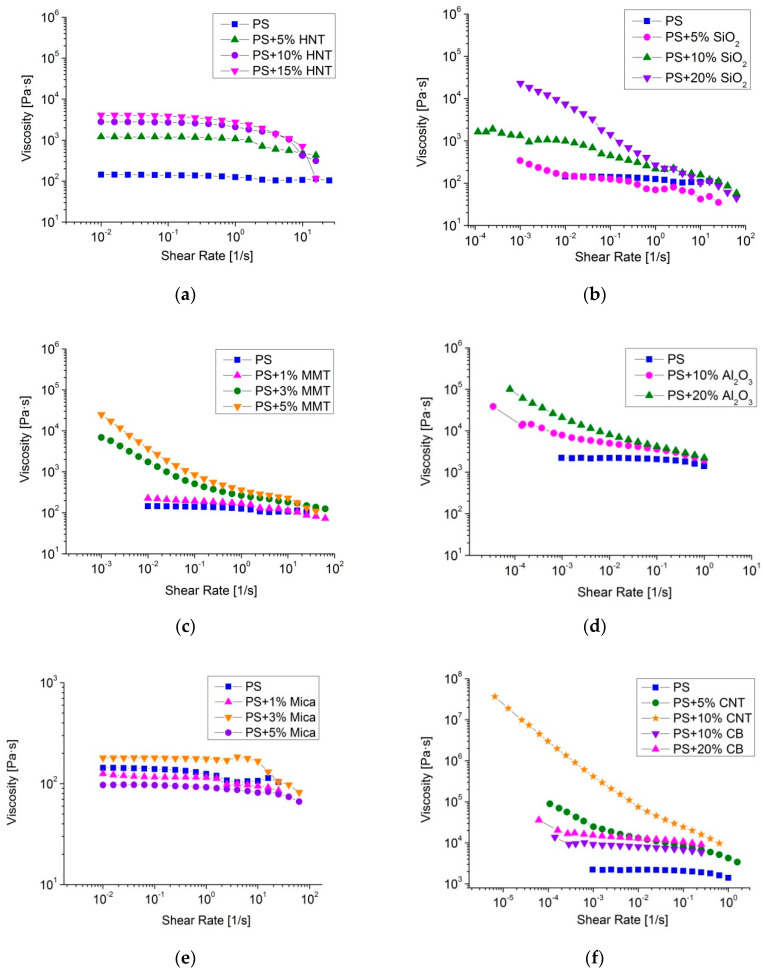
Experimental dependencies of viscosity on shear rate for pure PS and PS-based composites filled with: halloysite natural tubules at 220 °C (**a**); silicon dioxide at 220 °C (**b**); montmorillonite (MMT) at 220 °C (**c**); alumina nanoparticles at 200 °C (**d**); mica at 220 °C (**e**) and carbon nanoparticles at 200 °C (**f**).

Figure 2 presents experimental dependencies of melt viscosity on the shear rate for the fabricated PS-based composites with different filler concentrations. As can be seen in Figure 2f, the introduction of carbon nanoparticles caused an increase in melt viscosity, with the highest rise observed for filler particles with high aspect ratio (CNT). The viscosity of composites with 10% CB was higher than that of pure PS by an order of magnitude, while that of composites with 10% CNT was higher by two orders of magnitude at low shear rates. PS-based composites filled with MMT and Al_2_O_3_ (Figure 2c,d) showed similar dependencies: at maximal filler concentrations, 20% Al_2_O_3_ and 5% MMT, the melt viscosity at low shear rates increased by two orders of magnitude as compared to that of pure polymer. Composites filled with HNT and SiO_2_ also demonstrated increased melt viscosity, with the highest rise by an order of magnitude observed at low shear rates for samples with maximal filler concentrations, 15% HNT and 20% SiO_2_, see Figure 2a,b. The melt viscosity of composites with mica particles remained about the same as that of pure PS (Figure 2e).

**Figure 3:**
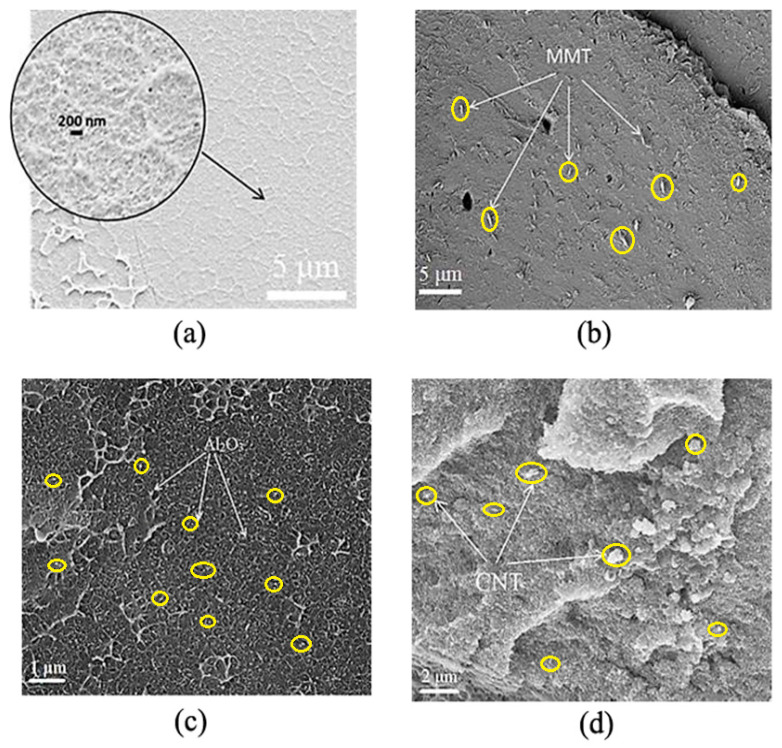
Microphotographs of cryo-cleaved surfaces of samples: (**a**) pure PS, (**b**) PS + 3% MMT, (**c**) PS + 10% Al_2_O_3_, (**d**) PS + 10% carbon nanotubes (CNT). Some filler particles and aggregates are depicted by arrows and yellow circles.

Therefore, the introduction of different types of particles, except Mica, to PS melts caused noticeable rise of melt viscosity depending upon filler concentration. The most pronounced rise was observed with the introduction of MMT, CNT and Al_2_O_3_ at high concentrations. The effective viscosity of the polymer melt at these filler concentrations increased by an average of 2 to 5 orders of magnitude over the pure PS. The rise of melt viscosity at low shear rates is known to be indicative of formation of a percolation network of filler particles in a polymer matrix, see [45,46]. In the case of mica, apparently the amount of filler used was insufficient to form such a network. Similar behavior of melts of mica-filled polymer composites has been observed in [47]. The rise of melt viscosity can be also partly due to formation of aggregates that impede the melt flow causing rise in its viscosity. For instance, agglomerates up to 1 μm in size have been observed in the PS + 10% CNT composite (see Figure 3). This composite also showed an increase in melt viscosity by 5 orders of magnitude over the pure PS (see Figure 2f).

### 3.2. Filler Distribution in the Polymer Matrix

The dispersion of filler particles in the polymer matrix was controlled by microscopic analysis of cryo-cleaved surfaces of composites. Representative examples of microphotographs of composites containing 3% MMT, 10% Al_2_O_3_ and 10% CNT are shown in Figure 3. As can be seen in Figure 3, all the three types of particles were rather uniformly distributed in the PS matrix, while agglomerates with a maximal size of about 1–2 μm were observed in some samples.

### 3.3. Linear Elastic Properties of Composites

**Figure 4:**
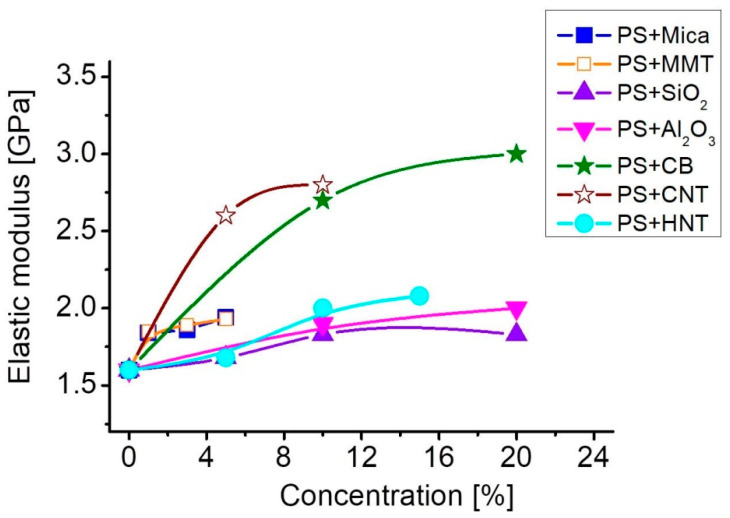
Elastic modulus of PS-based composites as function of filler concentration.

The values of elastic modulus, strength and strain at break of fabricated samples of PS-based composites filled with different nanoparticles are summarized in Table 3. The dependence of the elastic modulus of composites on the type and concentration of fillers is presented in Figure 4.

As can be seen from Table 3, the introduction of mica nanoparticles led to an increase in the elastic modulus of the PS-based composite reaching *E* = 1.9 GPa at the maximal concentration of 5%. That amounts for 19% rise over that for pure PS (*E*_0_ = 1.6 GPa). At the same time, the elongation at break reduced by 30% and reached a value of 3.9%. It is worth noting that composite strength remained at the level of pure PS (*σ*_b_ = 56 MPa) regardless of mica concentration. The introduction of HNT also caused an increase of the elastic modulus, however at higher filler concentrations. The noticeable rise of the modulus, by 25% and 30%, was achieved at, respectively, 10% and 15% concentrations of HNT. The elongation at break of these composites decreased by 60% and the strength at break by almost 20% as compared to pure PS. MMT nanoparticles provided changes in the elastic modulus similar to those with mica at the same concentrations. While strength and elongation at break demonstrated more profound changes: at the concentration of 5% MMT the sample strength decreased from 56 to 46 MPa (18%), and elongation decreased from 5.6% to 2.6% (54%). Thus the introduction of different alumosilicate particles into the PS matrix can provide noticeable increase in elastic modulus with just small decrease of strength at break.

Silicon oxide particles provided only slight increase in material rigidity at the concentrations up to 10%, while concentrations over 10% did not cause any further significant rise of the elastic modulus that achieved a maximum value of 1.83 GPa (14% rise over pure PS). With increasing stiffness, samples became much more brittle, their deformation at break *ε*_b_ dropped by 60% and achieved the value of 2.2% at 20% concentration of SiO_2_. The sample strength also decreased by about 50% at the concentrations of 10% and 20% SiO_2_. Introduction of spherical nanoparticles of Al_2_O_3_ resulted in the increase of material stiffness in tension. The elastic modulus rose up to 2 GPa (20% rise) at the filler concentration of 20%. At the same time, the composite strength and strain at beak decreased by 20% and 40%, respectively. Thus, the introduction of small spherical particles did not provide any challenging improvement of elastic properties of the material.

The introduction of carbon nanoparticles caused the most pronounced changes in elastic properties of composites. In particular, stiffness in tension of PS-based composites filled with CB increased noticeably and at the concentration of 10% the elastic modulus reached *E*_0_ = 2.8 GPa, that is 65% higher than that of pure PS. However higher filler concentrations did not cause any significant increase of the elastic modulus, which rose to *E*_0_ = 3.0 GPa only at 20% CB. With increasing stiffness of the samples, deformation at break decreased and at the concentration of 20% *ε*_b_ comprised 2.1%, which is 2.5 times lower than that of pure PS. The strength at break reached its maximum of 74 MPa at 10% CB, rising by 17% over that of pure PS and then dropped to 65 MPa at 20% CB being, however, still higher than that of pure PS. The introduction of CNT also provided an increase in the elastic modulus of composites, which achieved 2.8 GPa at 10% concentration. At the same time particles with high aspect ratio caused the decrease in both composite strength and strain at break. At 10% concentration of CNT *σ*_b_ = 49 MPa, that is 12% lower than that of pure PS and *ε*_b_ = 1.7%, that is more than 3 times lower than that of pure PS.

The behavior of elastic modulus of composites plotted in Figure 4 as a function of filler concentration demonstrates that the introduction of more rigid particles leads to a more profound increase in the elastic modulus, with the highest rise obtained with carbon nanofillers. The elastic modulus rose from 1.6 GPa for pure PS to 2.8 GPa for composites with 10% of CNT or CB. It should be emphasized that CB particles provided higher strength and strain at break of composites than CNT, with strength at break being even higher than that of pure PS. We can thus conclude that from the point of view of linear elasticity, relatively rigid carbon nanofillers provide more pronounced changes in elastic properties, with CB seeming more promising. This is in agreement with the fact that rigid carbon black aggregates introduce higher enhancement of the elastic modulus as compared to flexible aggregates [48]. However, the presence of agglomerates leads also to increased embrittlement of the samples, which we usually try to avoid.

### 3.4. Non-Linear Elastic Properties

The analysis of non-linear elastic properties of fabricated composite samples was performed for samples from each group of nanofillers, which demonstrated most profound changes of linear elastic properties: PS + 20% SiO_2_, PS + 10% HNT and PS + 20% CB. The analysis was based on measurements of the third-order elastic moduli and was examined in experiments on monitoring of the evolution of bulk non-linear strain waves in these composites.

#### 3.4.1. Measurements of the Third-Order Elastic Moduli

Table 4 presents sets of data on the second- and third-order moduli for 3-plate layered sandwiches of these composites, obtained from ultrasonic measurements. For comparison measurements were also performed on a bulk non-layered specimen made of commercial grade pure polystyrene. As can be seen from Table 4 elastic parameters of the specimen made of adhesively bonded plates of pure polystyrene fabricated by melt technology differ from those of commercial grade bulk specimen only slightly, and can be due to several factors, among which are the difference in polymer structure and fabrication process and the influence of adhesive layers. At the same time as can be readily seen from the Table all the three nanofillers cause more noticeable changes in both linear and non-linear elastic moduli. The noticeable rise of the second-order Lamé moduli *λ* and *μ* is observed in all composites The Young’s modulus *E* calculated from these data comprised 3.85 GPa for PS pure and 4.23, 4.29 and 4.58 GPa for nanocomposites with SiO_2_, HNT and CB particles, respectively. Thus in terms of linear elastic properties the addition of studied nanofillers to the polystyrene matrix provided efficient reinforcement of the material. The Young’s modulus *E* of composites obtained by ultrasonic measurements exceeded the static one (Table 3) by approximately 2 GPa. This corresponds to a typical frequency dependence of elastic moduli of polymer materials [49].

Changes in the non-linear, third-order moduli *l*, *m* and *n*, demonstrate more complex behavior. In general for all the composite samples changes in the non-linear moduli were more profound than changes in the linear, second-order moduli *λ* and *μ*. Among the non-linear moduli, the *l* modulus demonstrated prominent variations in all the composites with the maximal change of about 84% observed in the CB-containing one. The *n* modulus showed high relative variation, of about 91% only in PS + HNT samples, while variations of the *m* modulus were not so high and reached about 50% in PS + HNT and PS + CB nanocomposites.

#### 3.4.2. Evolution of Bulk Strain Solitons

The effect of changes in elastic features of polystyrene provided by the addition of different nanofillers was examined on the particular example of non-linear elastic process: formation and evolution of bulk non-linear strain waves in two- or three-layered waveguides containing one or two nanocomposite layer(s), respectively. In common with ultrasonic measurements, experiments were performed with the three types of fabricated composites: PS + 20% SiO_2_ particles, PS + 10% HNT and PS + 20% CB, which demonstrated the most profound change of the elastic modulus among each type of fillers (see Table 3). Soliton parameters obtained in waveguides with composite layers were compared with those obtained in waveguides of the same construction but with layers of pure polystyrene instead of composites.

**Figure 5:**
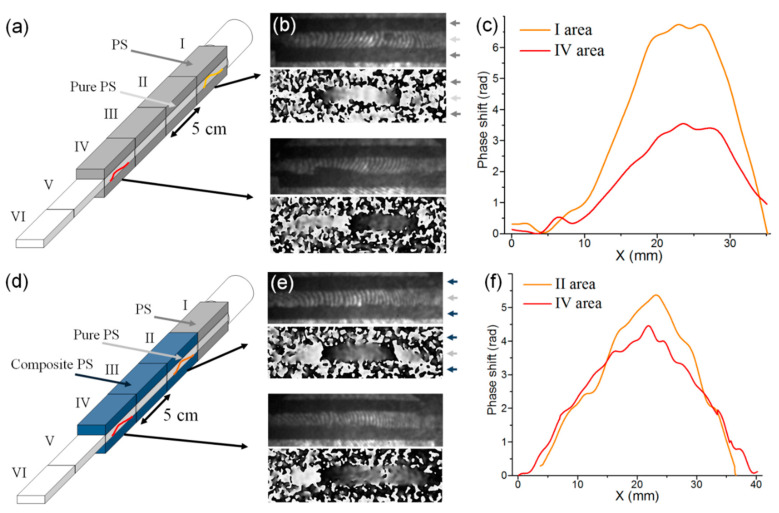
Holographic recording of solitons propagating in 3-layered waveguides made of pure PS (**a**–**c**) and with layers of nanocomposite PS + 10% HNT (halloysite natural tubules) (**d**–**f**). (**a**,**d**) waveguide schematics; (**b**,**e**) digital holograms and reconstructed phase images of solitons in the beginning (top rows) and at the end (bottom rows) of layered structures; (**c**,**f**) phase shift distributions in corresponding solitons.

Figure 5 presents waveguide schematics for the 3-layer layout, representative examples of recorded digital holograms and reconstructed phase images obtained in waveguides with HNT-containing composite layers and phase shift distributions representing solitons in the transparent middle layer.

Since the soliton is essentially a long trough-shaped wave with smooth fronts, its width is measured with relatively high error. Therefore, our analysis was based on the data on soliton velocity (which is directly related to its amplitude) and decay decrement. Taking into account the direct proportionality between the strain wave amplitude and recorded phase shift, the decay decrement was calculated as:

(7) $$\alpha =\frac{1}{x_{2}-x_{1}}ln\frac{\varphi_{1}}{\varphi_{2}}$$

where *x*_1_ and *x*_2_ are positions of soliton maxima in the two areas of the waveguide (for example, in areas I and IV in Figure 5a and in areas II and IV in Figure 5d).

The data obtained on soliton parameters in the aforementioned waveguides are summarized in Table 5. As can be seen from the Table, silica nanoparticles caused small modifications to the soliton parameters that are almost within the experimental errors. HNTs provided more noticeable changes, especially in terms of the decreased decay decrement. The addition of CB particles resulted in the most profound changes in soliton parameters. The general trend of these changes correlates with changes of elastic modulus obtained for composites with these fillings from tensile tests (Table 3). Furthermore, the non-linearity modulus *β*_s_ (Equation (6)), calculated from the data on linear and non-linear elastic moduli obtained from ultrasonic measurements (see Table 4), demonstrated a similar trend, showing the least change in SiO_2_-containing composites, and more significant changes in composites with HNT and CB particles.

## 4. Conclusions

We have performed a comprehensive analysis of mechanical properties of PS-based nanocomposites filled with different types of inclusions: spherical particles (SiO_2_ and Al_2_O_3_), alumosilicates (montmorillonite, halloysite natural tubules and mica), and carbon fillers (carbon black and multi-walled carbon nanotubes). The analysis was performed on composite samples fabricated under the same conditions and comprised tests at all steps of material fabrication procedure followed by assessment of linear elastic properties using standard measurement technologies and of non-linear elastic parameters using the laboratory set-up. The composites’ behavior was finally evaluated by monitoring the evolution of non-linear waves in them.

Testing of composite melts showed that introduction of all types of particles, except mica, to PS melts caused a noticeable rise of melt viscosity being more profound at higher filler concentration. The most pronounced rise was observed with the introduction of MMT, CNT and Al_2_O_3_ at high concentrations. At low shear rates the effective viscosity of polymer melts at these filler concentrations increased by an average of 2 to 5 orders of magnitude over pure PS, that is known to be indicative of a percolation network of filler particles in a polymer matrix.

The control of filler distribution in the polymer matrix verified that particles of all types were sufficiently uniformly distributed in the polymer matrix, and agglomerates sized no bigger than 1–2 μm have been observed in some samples at higher filler concentrations.

The analysis of linear elastic properties of fabricated samples demonstrated that the introduction of more rigid particles led to a more profound increase in the elastic modulus, with the highest rise of about 80% obtained with carbon fillers. It is worth noting that along with the enhancement of elastic modulus, CB particles provided also enhanced strength at break, about 20% higher than that of pure PS, and CNT particles allowed for maintaining this value about the same as that of pure PS.

Measurements of non-linear elastic parameters demonstrated that the third-order elastic moduli are more sensitive to the addition of filler particles to the polymer matrix than the second-order ones. However, no general trend was observed in their variations from one filler to another. Alternatively, the non-linearity modulus *β*_s_ used for description of bulk non-linear strain waves and comprising the combination of linear and non-linear elastic moduli of a material demonstrated considerable changes correlating with changes of the Young’s modulus in these composites. The absolute value of *β*_s_ showed the highest rise of 1.6 times in the HNT-containing composite as compared to that in pure PS. The changes in non-linear elasticity of fabricated composites were validated by measurements of the velocity and decay decrements of bulk non-linear strain waves. It was shown that the higher the rise in *β*_s_ value, the more significant was the increase of wave velocity and decrease of its decay decrement. We note that to the best of our knowledge this is the first fully-featured research into the complete set of linear and non-linear elastic properties performed for polymer nanocomposites with different types of filler particles.

## Figures and Tables

**Table 1:** Characteristics of 585 polystyrene.

Parameter	Value
Melt flow index	2.8 ± 0.7
Vicat softening temperature, °C	100
Tensile strength at break, MPa	48.0
Flexural strength, MPa	95.0
Weight fraction of residual styrene, %	0.05

**Table 2:** Dimensional specifications of filler particles.

Filler	Particle Size
SiO_2_	Diameter ~7 nm
Al_2_O_3_	Diameter ~13 nm
CB	Diameter ~80 nm
CNT	Diameter 10–40 nm, Length 1–25 μm
HNT	Diameter ~100 nm, Length 0.5–1.2 μm
Mica	Average size 1–5 *μ*m
MMT	Average size ≤ 10 *μ*m

**Table 3:** Mechanical properties of PS-based composites with different fillings.

Sample	Strength at Break *σ*_b_, MPa	Tensile Elastic Modulus *E*, GPa	Strain at Break *ε*_b_, %
PS pure	56 ± 1	1.6 ± 0.1	5.6 ± 0.2
PS + 1% Mica	56 ± 4	1.84 ± 0.02	4.4 ± 0.1
PS + 3% Mica	54 ± 2	1.86 ± 0.04	4.1 ± 0.2
PS + 5% Mica	56 ± 5	1.94 ± 0.05	3.9 ± 0.3
PS + 5% HNT	52 ± 1	1.68 ± 0.15	5.7 ± 0.3
PS + 10% HNT	49 ± 6	2.00 ± 0.06	2.8 ± 0.5
PS + 15% HNT	46 ± 4	2.08 ± 0.09	2.4 ± 0.2
PS + 1% MMT	58 ± 4	1.85 ± 0.04	3.9 ± 0.4
PS + 3% MMT	50 ± 4	1.89 ± 0.04	2.9 ± 0.3
PS + 5% MMT	46 ± 2	1.93 ± 0.07	2.6 ± 0.2
PS + 5% SiO2	52 ± 2	1.68 ± 0.15	5.7 ± 0.3
PS + 10% SiO2	32 ± 1	1.83 ± 0.17	2.8 ± 0.2
PS + 20% SiO2	31 ± 3	1.83 ± 0.2	2.2 ± 0.2
PS + 10% Al2O3	57 ± 1	1.9 ± 0.1	4.1 ± 0.5
PS + 20% Al2O3	49 ± 1	2.0 ± 0.1	3.1 ± 0.2
PS + 10% CB	74 ± 2	2.8 ± 0.1	3.1 ± 0.2
PS + 20% CB	65 ± 5	3.0 ± 0.2	2.1 ± 0.6
PS + 5% CNT	53 ± 3	2.6 ± 0.2	1.9 ± 0.3
PS + 10% CNT	49 ± 3	2.8 ± 0.2	1.7 ± 0.2

**Table 4:** Second- and third-order elastic moduli of nanocomposite samples determined from ultrasonic measurements. Second-order moduli *λ* and *μ* are measured within error tolerance of 0.02 and 0.01 GPa, respectively.

Material	*ρ,* g/cm^3^	Elastic Moduli, GPa					Nonlinearity Modulus *β_s_*, GPa
		*λ*	*μ*	*l*	*m*	*n*	
PS commercial	1.05	2.80	1.44	−46.2 ± 1.8	−14.8 ± 0.7	−7.5 ± 0.6	−32.6
PS pure	1.00	2.62	1.37	−42.2 ± 1.3	−12.1 ± 0.5	−5.5 ± 0.4	−25.3
PS+20%SiO_2_	1.16	3.28	1.55	−62.9 ± 1.9	−15.5 ± 0.9	−7.1 ± 0.7	−32.3
PS+10%HNT	1.10	2.95	1.59	−40.0 ± 2.1	−18.2 ± 1.0	−10.7 ± 0.9	−42.3
PS+20%CB	1.21	3.72	1.78	−85.3 ± 2.8	−19.6 ± 0.8	−5.6 ± 0.8	−40.8

**Table 5:** Soliton velocities and decay decrements in nanocomposites.

Material	Soliton Velocity *υ*, m/s	Decay Decrement, *α*, cm^−1^
PS commercial	1800 ± 7	0.012 ± 0.006
PS pure	1772 ± 10	0.041 ± 0.006
PS + 20% SiO_2_	1801 ± 9	0.039 ± 0.006
PS + 10% HNT	1820 ± 10	0.018 ± 0.004
PS + 20% CB	1887 ± 13	0.010 ± 0.005
